# Unintended Laboratory-Driven Evolution Reveals Genetic Requirements for Biofilm Formation by *Desulfovibrio vulgaris* Hildenborough

**DOI:** 10.1128/mBio.01696-17

**Published:** 2017-10-17

**Authors:** Kara B. De León, Grant M. Zane, Valentine V. Trotter, Gregory P. Krantz, Adam P. Arkin, Gareth P. Butland, Peter J. Walian, Matthew W. Fields, Judy D. Wall

**Affiliations:** a Department of Biochemistry, University of Missouri, Columbia, Missouri, USA; b Environmental Genomics and Systems Biology Division, Lawrence Berkeley National Laboratory, Berkeley, California, USA; c Department of Microbiology and Immunology, Montana State University, Bozeman, Montana, USA; d Center for Biofilm Engineering, Montana State University, Bozeman, Montana, USA; e Department of Bioengineering, University of California, Berkeley, California, USA; f Molecular Biophysics and Integrated Bioimaging Division, Lawrence Berkeley National Laboratory, Berkeley, California, USA; University of Washington

**Keywords:** *Desulfovibrio vulgaris*, biofilms, genetic polymorphisms, secretion systems, sulfate reduction

## Abstract

Biofilms of sulfate-reducing bacteria (SRB) are of particular interest as members of this group are culprits in corrosion of industrial metal and concrete pipelines as well as being key players in subsurface metal cycling. Yet the mechanism of biofilm formation by these bacteria has not been determined. Here we show that two supposedly identical wild-type cultures of the SRB *Desulfovibrio vulgaris* Hildenborough maintained in different laboratories have diverged in biofilm formation. From genome resequencing and subsequent mutant analyses, we discovered that a single nucleotide change within DVU1017, the ABC transporter of a type I secretion system (T1SS), was sufficient to eliminate biofilm formation in *D. vulgaris* Hildenborough. Two T1SS cargo proteins were identified as likely biofilm structural proteins, and the presence of at least one (with either being sufficient) was shown to be required for biofilm formation. Antibodies specific to these biofilm structural proteins confirmed that DVU1017, and thus the T1SS, is essential for localization of these adhesion proteins on the cell surface. We propose that DVU1017 is a member of the *lapB* category of microbial surface proteins because of its phenotypic similarity to the adhesin export system described for biofilm formation in the environmental pseudomonads. These findings have led to the identification of two functions required for biofilm formation in *D. vulgaris* Hildenborough and focus attention on the importance of monitoring laboratory-driven evolution, as phenotypes as fundamental as biofilm formation can be altered.

## IMPORTANCE

The growth of bacteria attached to a surface (i.e., biofilm), specifically biofilms of sulfate-reducing bacteria, has a profound impact on the economy of developed nations due to steel and concrete corrosion in industrial pipelines and processing facilities. Furthermore, the presence of sulfate-reducing bacteria in oil wells causes oil souring from sulfide production, resulting in product loss, a health hazard to workers, and ultimately abandonment of wells. Identification of the required genes is a critical step for determining the mechanism of biofilm formation by sulfate reducers. Here, the transporter by which putative biofilm structural proteins are exported from sulfate-reducing *Desulfovibrio vulgaris* Hildenborough cells was discovered, and a single nucleotide change within the gene coding for this transporter was found to be sufficient to completely stop formation of biofilm.

## INTRODUCTION

*Desulfovibrio vulgaris* Hildenborough is a sulfate-reducing bacterium (SRB) for which genetic manipulation has been established (1). SRB, often found attached to surfaces as a biofilm, can be beneficially utilized in industrial processes, including removal of sulfates and metals in wastewater treatment (2), bioremediation of toxic metals in hazardous waste sites (3, 4), and production of energy in microbial fuel cells (5). In contrast, these robust qualities cause these biofilms to be exceedingly problematic in many industrial processes, causing clogging of pipelines, souring of products with metabolic by-products, and corrosion of ferrous metals and concrete. In 1994, the direct loss from corrosion in U.S. industry was estimated to be $300 billion, of which 20% was attributed to microbially related processes (6). SRB are the most common culprit in anaerobic microbially influenced corrosion, and their biofilms accelerate the problem by allowing for locally high concentrations of corrosive metabolites (7).

Yet, the mechanism of biofilm formation of SRB such as *D. vulgaris* Hildenborough has not been determined. Studies of SRB biofilm on steel have predominantly focused on the exopolysaccharide fraction of the biofilm matrix, where targeted and genome-wide expression analyses have shown increases in expression of exopolysaccharide biosynthesis proteins in biofilm compared to planktonic cells (8, 9). The matrix of *D. vulgaris* Hildenborough biofilm on glass slides was observed to be predominantly comprised of protein (10), with a carbohydrate/protein (C/P) ratio of the biofilm biomass of approximately 0.13 µg/µg (11). The proteins encoded in DVU1012 and DVU1545 were reported to be prevalent in the extracellular fraction from *D. vulgaris* Hildenborough biofilms on glass slides (11). These uncharacterized large proteins (3,038 and 2,414 amino acids, respectively) are annotated as hemolysin-type calcium-binding repeat proteins. While both proteins contain calcium binding domains and glycine-rich repeats, the protein encoded by DVU1012 also has a von Willebrand factor A domain, which has been shown to be involved in cell-cell attachment in eukaryotic cells (12). Two other proteomic studies of *D. vulgaris* Hildenborough in planktonic cultures (one of outer membrane proteins and one of abundant, large proteins) both identified the protein encoded in DVU1012 in their studies; however, neither identified the protein encoded in DVU1545 (13, 14). This suggests a difference in abundance for these proteins. Interestingly, neither of the transcriptomic studies of *D. vulgaris* Hildenborough biofilms on glass or mild steel reported significant changes in expression for DVU1012 or DVU1545 in comparison with transcripts from planktonic cells (9, 11). Thus, to our knowledge, with the exception of suggesting a role in extracellular matrix formation (11), the function of these proteins had not been characterized.

While establishing biofilm reactors in our laboratory following protocols described previously and using the same bacterial strain (11), we discovered that our *D. vulgaris* Hildenborough strain was deficient in biofilm formation. This unexpected result serendipitously led to the identification of two functions required for biofilm formation by *D. vulgaris* Hildenborough.

## RESULTS

**FIG 1:**
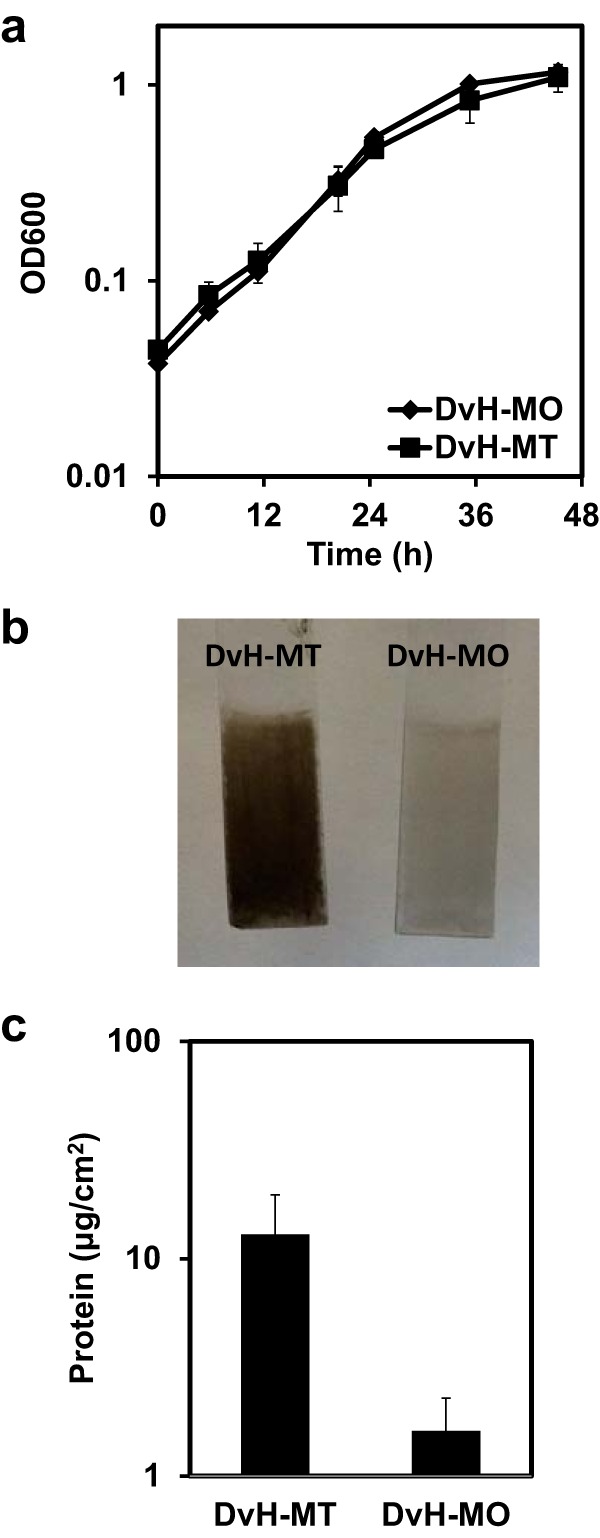
Growth and batch biofilm comparison of DvH-MO and DvH-MT. (a) Growth of DvH-MO (diamonds) and DvH-MT (squares) in LS4D medium at 30°C. Error bars denote standard deviations from the average from triplicate samples. (b) To compare biofilms, anaerobic cultures in Balch tubes containing LS4D with a halved glass microscope slide were inoculated and incubated for 48 h. The slides were removed and imaged. (c) Biofilm was measured as protein, scraped from the glass slides. Error bars denote the standard deviations from two biological and two technical replicates.

**FIG 2:**
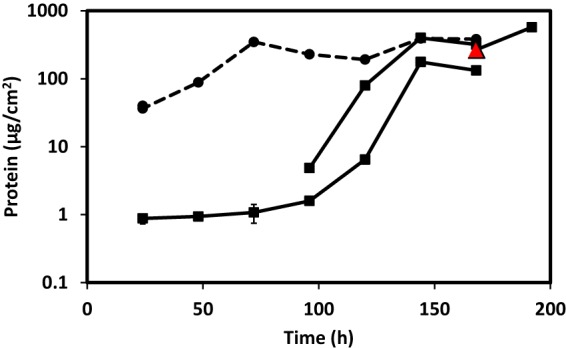
Biofilm reactors of DvH-MO and DvH-MT. Biofilms of DvH-MO (solid lines) and DvH-MT (dashed line) from reactors were scraped from halved glass microscope slides sampled at intervals and measured by protein. The limit of detection was 1.3 μg protein/cm^2^. Time 0 denotes the time at which the cultures were transitioned from batch to continuous growth in order to control the growth rate by substrate availability. Two representatives of four DvH-MO reactor runs are displayed. Error bars denote the standard deviations from three technical replicates; most are within the symbols. The red triangle (168 h) indicates the biofilm sample collected for DvH-MO genome resequencing. Two biofilm samples were collected at 24 h for DvH-MT and are nearly overlapping.

Upon setting up the biofilm reactor system used by the laboratory of Matthew Fields at Montana State University (11), we could not recapitulate biofilm growth kinetics with the “identical” strain of *D. vulgaris* Hildenborough in our laboratory at the University of Missouri. The *D. vulgaris* Hildenborough strain maintained at Montana State University (DvH-MT) was received and compared directly with the strain housed at the University of Missouri (DvH-MO). Despite similar planktonic growth rates (Fig. 1a), when biofilms were grown in batch cultures containing halved glass microscope slides as a surface substrate, approximately eight times more biofilm was formed by DvH-MT in 48 h (Fig. 1b and c). Furthermore, when grown in biofilm reactors, DvH-MT initiated biofilm within 24 h and reached steady-state biofilm (i.e., when cells incorporated into biofilm via growth or attachment equal cells lost from detachment resulting in no net gain of biofilm as measured as protein on surface over time) within 70 h, similar to the observations previously reported for this strain (11) (Fig. 2). In contrast, DvH-MO consistently lagged in biofilm formation for approximately 100 h before reaching a detectable limit of 1.3 μg protein/cm^2^ (Fig. 2), despite having planktonic optical density at 600 nm (OD_600_) measurements near 1.0 in the reactors, comparable to those of DvH-MT. Yet when steady-state biofilm was achieved, the biofilm quantities were not different.

**FIG 3:**
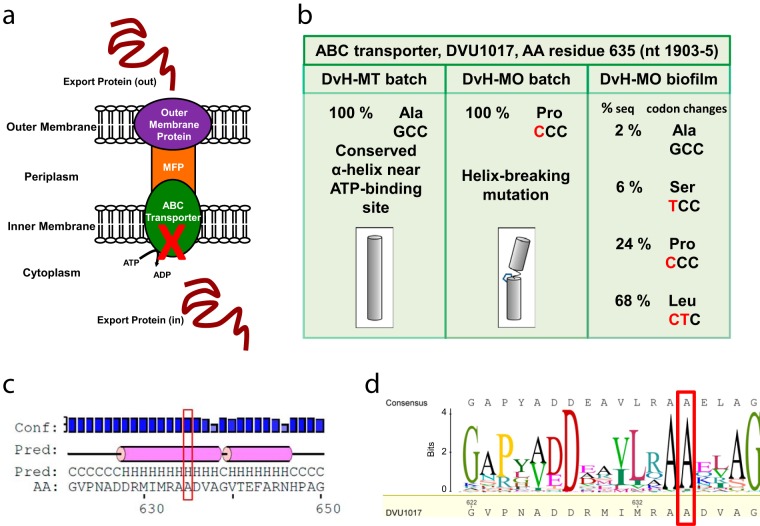
DvH-MO SNP mutation in a conserved α-helix of the ABC transporter DVU1017 of a putative type I secretion system (T1SS). (a) Cartoon representation of a T1SS complex comprised of three subunits: the outer membrane protein, the membrane fusion protein (MFP), and the ABC transporter (16). The red X marks the subunit with the SNP found in planktonic DvH-MO. (b) Sequence and amino acid changes found at the 635th residue of the DVU1017 protein, the predicted ABC transporter. Shown is a cartoon representation of the possible proline kink in this predicted α-helix and the change in direction that the adjacent amino acids are facing (depicted by shading). (c) Secondary structure prediction of the 622- to 650-amino-acid region of DVU1017 that encompasses residue 635. Prediction was with PSIPRED. Pink cylinders denote a helical structure, H, that is flanked by coils, C. The PSIPRED confidence of the prediction is shown along the top. (d) A sequence logo generated from an alignment of the DVU1017 amino acid sequence to the top 475 BLAST hits, excluding other *Desulfovibrio* spp., shows the conservation of Ala at 635. The frequency of amino acid is depicted by the size of the letter in bits. The red box highlights Ala_635_ in both panels c and d.

We hypothesized that an unintended mutation had occurred in one of the two strains to alter the biofilm formation capacity. To test this, the genomes were resequenced at an average of 63× coverage for DvH-MT and DvH-MO in batch cultures and DvH-MO biofilm at steady state (168 h). Because DvH-MO formed a biofilm after a significant delay, we hypothesized that the cells finally found in the biofilm would show gene changes that could restore this function to the culture. In all three samples, there were 29 deviations from the published sequence that are likely errors in the original genome sequencing (15) (see Table S1 in the supplemental material). Whereas 12 mutations were found to be unique to DvH-MO batch cultures at or near 100% frequency (Table 1), no unique mutations were found in DvH-MT at ≥50% frequency. Of note, a DvH-MO G1903C single nucleotide polymorphism (SNP) in DVU1017 resulted in an alanine-to-proline substitution at amino acid residue 635 in the encoded protein (Fig. 3). The levels of sequencing coverage of this region were 68-, 64-, and 50-fold, for the DvH-MT batch, DvH-MO batch, and DvH-MO biofilm, respectively. The gene at DVU1017 was annotated as encoding the ABC transporter of a type I secretion system (T1SS). A T1SS is comprised of three subunits (Fig. 3a): the inner membrane ABC transporter, the periplasmic membrane fusion protein, and the outer membrane pore protein (16). As these systems transport proteins to outside the cell (16) and the *D. vulgaris* Hildenborough biofilm matrix is predominantly protein (10), it was plausible that a mutation in this gene causing a defective transporter could prevent biofilm formation. Alignments of the DVU1017 protein to other ABC transporters, comparisons to known crystal structures (Protein Data Bank no. 2FF7 and 4S0F), and secondary structure prediction with PSIPRED (v3.3) (17) indicated that Ala_635_ was highly conserved and was located in an α-helix near the ATP-binding site (Fig. 3c and d). From comparisons of structures of transmembrane helices, proline in an α-helix was determined to cause a helix kink or distortion between the helical portions (18). The G1903C mutation in DVU1017 of DvH-MO resulting in the A635P substitution could alter the nucleotide binding pocket or prevent access to the binding site.

10.1128/mBio.01696-17.2 TABLE S1  Deviations from published sequence in both DvH-MO and DvH-MT are possible sequencing errors in the original sequencing. Download TABLE S1, DOC file, 0.1 MB. Copyright © 2017 De León et al. 2017 De León et al. This content is distributed under the terms of the Creative Commons Attribution 4.0 International license.

**TABLE 1:** Mutations in DvH-MO batch culture

Location^a^	Locus tag	Gene description/notes	Amino acid change	Nucleotide change
268940		29 bp upstream of DORF26459		A→T
468337		140 bp upstream of DORF27863		C→G
1034706	DVU0942	Ferric uptake regulator (fur)	K→Q	A→C
**1118956**	**DVU1017**	**ABC transporter, ATP-binding protein/permease protein**	**A→P**	**G→C**
1213311	DVU1107	Phage tail tape measure protein		G→A
1884231	DVU1819	Protein-export membrane protein SecD	P→A	G→C
2099891	DVU2020	Type I phosphodiesterase/nucleotide pyrophosphatase/phosphate transferase		G→A
2904348	DVU2801	Hypothetical protein	V→G	T→G
2918165		220 bp downstream of DVU2815 outer membrane efflux protein, 573 bp upstream of DVU2812 formate dehydrogenase		C→T
3098151	DVU2990	Molybdopterin biosynthesis MoeA protein	A→G	C→G
P_40,114	DVUA0034	Conserved domain protein; dinucleotide-utilizing enzyme involved in molybdopterin and thiamine biosynthesis	P→R	G→C
P_92,886	DVUA0070	Conserved domain protein; polysaccharide pyruvyl transferase		A→T

a Reference sequence NCBI GenBank accession no. NC_002937.3 and NC_005863.1. P_40,114 and P_92,886 indicate the positions on the plasmid (bp 40114 and 92886, respectively). The mutation characterized in this study has been highlighted in boldface.

If the DVU1017(G1903C) transversion were responsible for eliminating biofilm formation, it would be expected that a selected reversion or suppression of that mutation would be found in a majority of the sequences from DvH-MO biofilm. In genomic sequences obtained from DvH-MO biofilm, only 24% of sequencing covering this location contained the G1903C mutation. Instead, 2% matched the published sequence, 6% contained a G1903T mutation resulting in a codon change of A635S, and 68% contained a secondary mutation of GC1903CT resulting in a codon change of A635L (Fig. 3b). We hypothesized that the A635P was preventing biofilm formation in DvH-MO and that a secondary mutation in the majority of cells in the biofilm resulted in an A635L substitution that suppressed this inhibition and allowed biofilm to form.

**FIG 4:**
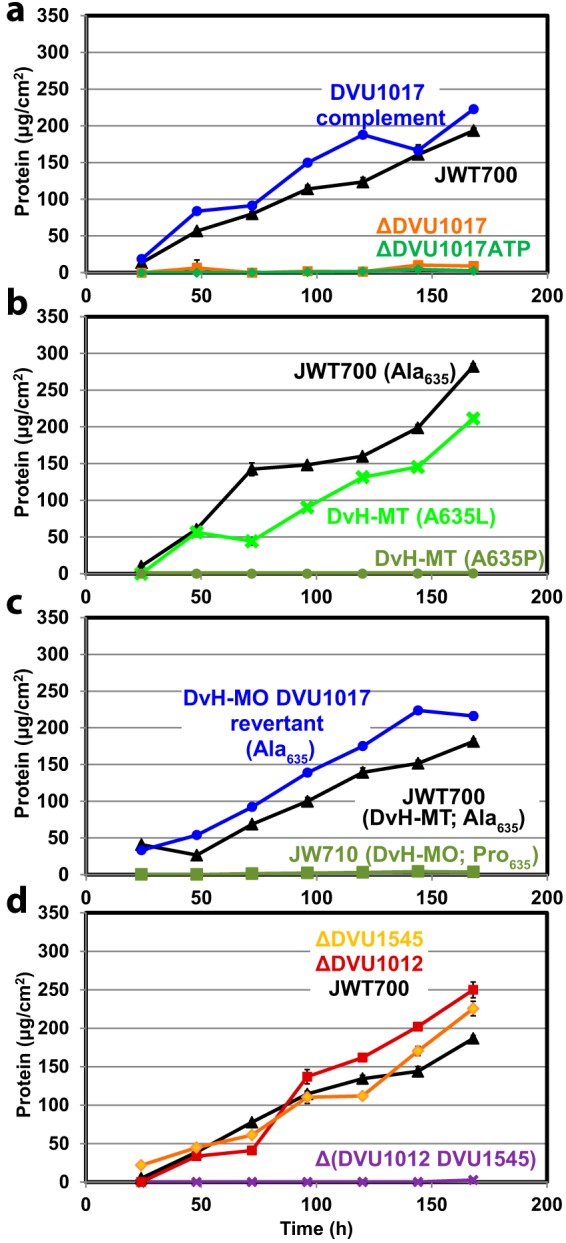
*D. vulgaris* Hildenborough biofilm formation in bioreactors of strains with altered DVU1017 or mutants deleted for predicted cargo proteins. Representative reactors are presented; replicated reactors are shown in Fig. S1. Time 0 denotes the time at which the cultures were transitioned from batch to continuous growth in order to control the growth rate by substrate availability. Biofilm of the parental strain JWT700 (DvH-MT Δupp) is shown as black triangles. (a) Biofilm formation by DvH-MT strains lacking the entire DVU1017 gene (orange squares), a mutant lacking the DVU1017 ATP-binding domain region (bp 1543 to 2331 [green diamonds]), or the latter strain complemented with DVU1017 (blue circles). (b) SNP mutations were introduced into JWT700, resulting in DVU1017 amino acid changes of A635P or A635L (dark green circles and light green X, respectively). (c) Biofilm formation by the DvH-MO DVU1017 Pro_635_ parental strain (JW710 Δ*upp* [dark green squares]), a DVU1017(P635A) revertant (blue circles). (d) Single and double mutants of the putative T1SS-secreted, biofilm structural proteins DVU1012 and DVU1545 (red squares and gold diamonds, respectively, and a purple X for the double mutant) were created in the DvH-MT background and tested for altered biofilm formation. Error bars denote the standard deviations from triplicate samples in the protein assay; most are within the symbols.

To test the essentiality of DVU1017 on biofilm formation, we deleted this gene from the DvH-MT parental strain JWT700 (Δ*upp* [see Table S2 in the supplemental material]). Deletion of this gene eliminated biofilm formation (Fig. 4a; see Fig. S1 in the supplemental material). Furthermore, deletion of only the ATP-binding domain of DVU1017 was sufficient to eliminate biofilm formation. Complementation of the gene region encoding the wild-type ATP-binding domain restored the biofilm phenotype similar to that of the parental strain (Fig. 4a). However, biofilm formation was not restored when the deletion strain was complemented with the region encoding the ATP-binding domain containing the DvH-MO mutation of A635P (Fig. 4b). By comparison, biofilm was restored with the introduction of the mutated gene encoding the A635L substitution within the ATP-binding domain (Fig. 4b). Thus, we deduced that the A635P mutation in DvH-MO caused the elimination of biofilm formation in this strain. Restoration of the biofilm phenotype by the A635S mutant was not tested. The ability of DvH-MO to form biofilm depends on sufficient incubation time for a small number of DVU1017(A635L) (and possibly A635S) mutants to become detectable and dominate the biofilm.

10.1128/mBio.01696-17.1 FIG S1  Replicate biofilm reactors of the DvH-MT parental strain and transporter and biofilm structural protein mutants. Shown are replicate reactors of those displayed in Fig. 4. (a) The DvH-MT parental strain JWT700 (Δ*upp*) was grown in each series of reactors as a control and to track consistency (black triangles [*n =* 6]). (b) The DvH-MT strains lacking the entire DVU1017 gene (orange squares) and single and double mutants of the putative T1SS-secreted, biofilm structural proteins DVU1012 and DVU1545 (red squares and gold circles, respectively, and purple “X” for double mutant) were created in the DvH-MT background. Download FIG S1, PDF file, 0.2 MB. Copyright © 2017 De León et al. 2017 De León et al. This content is distributed under the terms of the Creative Commons Attribution 4.0 International license.

10.1128/mBio.01696-17.3 TABLE S2  Strains and plasmids used in this study. Download TABLE S2, XLS file, 0.1 MB. Copyright © 2017 De León et al. 2017 De León et al. This content is distributed under the terms of the Creative Commons Attribution 4.0 International license.

Because of the expected low frequency of spontaneous mutation and the reproducibility of the lag in biofilm formation for DvH-MO, it was unlikely that a mutation allowing for biofilm formation was arising within the reactor. The lack of biofilm formation (during 168 h of continuous growth in the reactor) by the complemented construct containing the region of DvH-MO DVU1017 encoding the ATP-binding domain with the A365P alteration (Fig. 4c) supported this deduction. Instead, we hypothesized that suppressors and revertants were present in the DvH-MO inoculum at a low abundance, but were less than 2% of the population because they were not detectable in the 64× sequence coverage of this region. In the DvH-MO reactor from which the sample for reversion and suppression identification was collected, the initial biofilm detected had 4.5 µg protein/cm^2^ at 96 h of continuous growth (equating to 120 h of total time within the reactor after accounting for 24 h of initial batch growth) (Fig. 2). The available surface area within the reactor was approximately 336 cm^2^ (231 cm^2^ on the vessel and 15 cm^2^ for each glass slide). Assuming that the biofilm matrix made up a minor portion of the total protein, this protein content equates to ca. 5.4 × 10^9^ cells as biofilm with the additional assumption that there is 278 fg protein/cell, as was found in *Desulfovibrio desulfuricans* CSN (19). After accounting for the 24% of the biofilm population that were DVU1017(A635P) mutants, and thus could not make biofilm on their own, 4.1 × 10^9^ cells of the biofilm were considered biofilm producing. The generation time of DvH-MO was determined from batch cultures to be 6.8 h (Fig. 1a). Thus, in 120 h, 18 doublings had occurred. Therefore, in order to have 4.1 × 10^9^ biofilm-producing cells within the biofilm, there would need to have been at least 1.6 × 10^4^ biofilm-producing cells in the approximate 10^10^ cells of inoculum. A similar result of 9.9 × 10^3^ biofilm-producing cells in the inoculum was found when calculated based upon 398 µg protein/cm^2^ at 144 h after the batch cultivation (168 h of total time within the reactor). The actual number of biofilm-producing cells in the inoculum was likely larger, as these calculations are based upon the assumption that there was no loss of biofilm-producing cells in the effluent. This shows that a spontaneously biofilm-producing mutant arising within the reactor would not have had sufficient time to generate the amount of biofilm observed.

To ensure that the DVU1017(A635P) mutation was the only mutation in DvH-MO that contributed to the biofilm deficiency, DVU1017 was restored to Ala_635_ in the DvH-MO mutant parental strain, JW710 (Δ*upp*; DVU1017 with the A635P SNP). As a control, no biofilm formed within the duration of the reactor runs for the original parental strain (Fig. 4c). However, upon restoring the DVU1017 SNP back to G (encoding alanine), this strain formed biofilm similar to that of DvH-MT. This confirmed that the SNP causing the A635P substitution in DVU1017 and not any of the other 11 mutations was the sole reason for biofilm deficiency in DvH-MO.

As DVU1017 was annotated as an ABC transporter of a T1SS, we hypothesized that the A635P mutation was inhibiting the export of one or more proteins essential for biofilm formation. T1SS export proteins can include hemolysins, metalloproteases, and adhesins. Whereas, there is little conservation across the amino acid sequences of these cargo proteins, structural characteristics have allowed for putative identification (16). Only two proteins in *D. vulgaris* Hildenborough were identified that met the requirements of a T1SS-exported protein: those encoded by DVU1012 and DVU1545, both annotated as hemolysin-type calcium-binding repeat proteins (Table 2). These proteins had been identified previously as being present in the *D. vulgaris* Hildenborough biofilm matrix (11). To test whether these proteins were required for biofilm formation by *D. vulgaris* Hildenborough, each was deleted individually from the DvH-MT parental strain. Both single mutants were still able to form biofilm (Fig. 4d; Fig. S1). However, a double-deletion mutant was constructed and observed to be incapable of forming biofilm. These results were interpreted to demonstrate that at least DVU1012 or DVU1545 protein is required for biofilm formation and that these two proteins are compensatory under the conditions tested and given the assays used in this work. The biofilm by each single mutant appeared to have no major phenotypic differences, and the rates and amounts of biofilm formed were similar.

**TABLE 2:** T1SS export protein characteristics in DVU1012 and DVU1545

Characteristic	Characteristic in:	
	DVU1012	DVU1545
Large	3,038 amino acids	2,414 amino acids
Acidic	pI 4.03	pI 4.20
Few or no cysteines	0 cysteines	3 cysteines
Ca-binding recognition repeat^a^	Present (PS00330)	Present (PS00330)
Gly/Ala repeats	Present	Present
9-mer amino acid repeat near C terminus (GGXGXDXXX)^b^	GGsGdDtiv (2852–2860)	GGnGhDvld (2263–2271)
	GGlGnDtlg (2870–2878)	GGtGnDtlh (2272–2280)
	GGlGnDtii (2888–2896)	GGeGvDtil (2300–2308)
	GGaGtDtle (2916–2924)	

a The Prosite accession number is given in parentheses.

b Motif from Delepelaire (16). Capital letters represent conserved residues within the repeat. The positions of the repeats are shown in parentheses.

**FIG 5:**
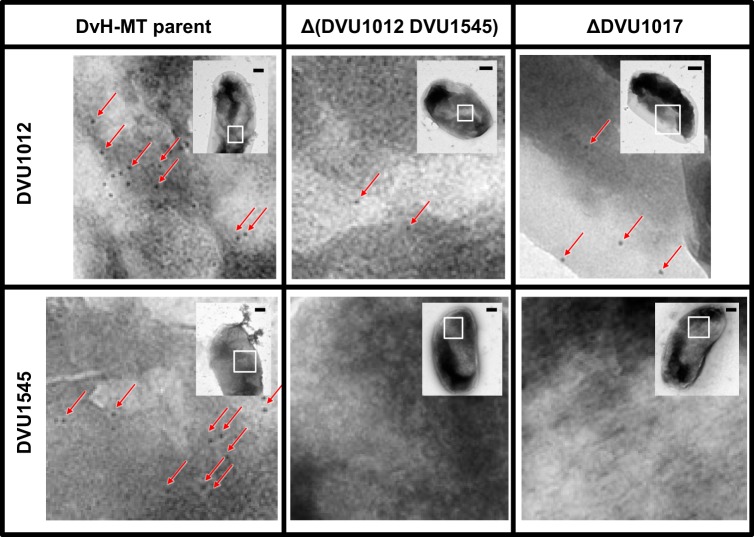
Electron microscopy images of DVU1012 (a) and DVU1545 (b) antibody-treated DvH-MT cells. Antibody binding of representative cells of the DvH-MT parental strain JWT700, the putative biofilm structural protein Δ(DVU1012 DVU1545) double mutant, and the ΔDVU1017 ABC transporter mutant. Binding is depicted by black spots of 10-nm-diameter gold particles, and examples are highlighted by red arrows. Images are enlargements of the white boxed region from the inset. Scale bars in the insets represent 200 nm.

To confirm that DVU1017 was required for localization of DVU1012 and DVU1545 on the cell surface, antibodies to DVU1012 and DVU1545 were generated and antibody binding to whole cells was compared between the DvH-MT parental strain and the constructed DVU1017 deletion mutant (Fig. 5). The ABC transporter mutant had little binding of either the DVU1012 or DVU1545 antibodies compared to the parental strain. To test for nonspecific binding, the double-deletion mutant of DVU1012 and DVU1545 was used as a control. Binding was similar between this double mutant and the DVU1017 mutant, regardless of which protein the antibody was targeting.

## DISCUSSION

Unintended laboratory evolution has resulted in the divergence of *D. vulgaris* Hildenborough strains in the ability to form biofilms. It is important to note that no directed evolution occurred for this strain. However, a culture is likely always under selection in laboratory culturing as researchers continuously try to optimize growth under conditions conducive for the laboratory environment. Routine culturing would select for planktonic cells as the biofilm may remain attached to the sides or settle to the bottom of the vessel. A uniform culture allows for ease of growth measurements and trait documentation; thus, a culture with these characteristics may be preferentially chosen for downstream application. The spontaneous mutation rate in *Escherichia coli* is ~10^−10^ per bp per replication (20). For a 3.5-Mb genome like that of *D. vulgaris* Hildenborough, this would equate to approximately 1 mutation per 7 generations. The probability of choosing one of these mutants when going through single-colony isolation would be exceedingly low. However, when a spontaneous mutation arises that results in greater fitness or easier manipulation characteristics compared to the wild-type strain in the culture, these mutants can become moderate or abundant in the population (21, 22). This increased abundance of a spontaneously occurring mutant increases the probability of fixing the mutation into the population through genetic bottlenecks such as choosing a single colony during strain isolation or during genetic manipulation. Consequently, these genetic changes can result in loss of phenotypes nonessential to routine laboratory cultivation.

It is likely that DvH-MO has gone through one or more single-colony selections since receipt in the lab in 2003. These bottlenecks apparently fixed 12 unique mutations in the DvH-MO strain compared to the published sequence. One of these mutations resulted in the codon change of A635P in DVU1017, the ABC transporter of a T1SS. Earlier studies by Hinsa et al. (23) established that a T1SS is required for biofilm formation in *Pseudomonas fluorescens*. Here we show that DVU1017, and thus the T1SS, is required for biofilm formation in *D. vulgaris* Hildenborough (Fig. 4a). To our knowledge, this is the first description of a T1SS required for biofilm formation in a strict anaerobe.

Furthermore, the single nucleotide change (G1903C) in DVU1017 resulting in A635P was sufficient to eliminate biofilm formation (Fig. 4b). Given selection in approximately 100 h of continuous growth, DvH-MO did form a biofilm in which a secondary mutation (C1904T) resulting in the A635L change in DVU1017 was dominant (Fig. 2 and 3). This mutation was not detected in the DvH-MO batch culture sequencing, and estimations of the number of cells within the biofilm at the point of detection are too high to be accounted for by a spontaneous SNP arising early in the reactor. DVU1017 was amplified and sequenced from a recently obtained culture of *D. vulgaris* Hildenborough from the American Type Culture Collection (ATCC), and mutations were not detected. These data support the premise that the DVU1017(A635L) strain within the DvH-MO “wild-type” culture was not generated within the reactor system but was present at a low abundance in the inoculum.

The two *D. vulgaris* Hildenborough genes (DVU1012 and DVU1545) found to encode proteins containing the recognition motif for export by the T1SS were clearly compensatory for biofilm production. The SNP in DVU1017 was likely impeding export of these two proteins, which through proteomic studies had previously been found to be abundant in the extracellular fraction of *D. vulgaris* Hildenborough biofilm (11). The scarcity of binding of antibodies either for DVU1012 or for DVU1545 to the DVU1017 mutant cell surface verifies that this ABC transporter is required for localization of DVU1012 and DVU1545 proteins on the cell surface (Fig. 5). No other genes in *D. vulgaris* Hildenborough encode a protein containing the calcium-binding domain that is the T1SS export recognition motif.

**FIG 6:**
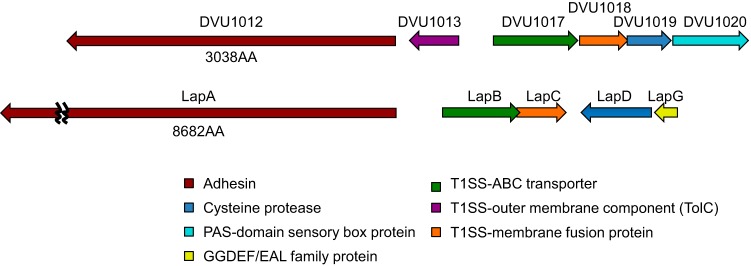
DvH ortholog arrangement (a) compared to the *lap* operon (b) of *Pseudomonas putida* KT2440. Arrows represent the relative size and position of the predicted coding regions, and the arrowhead represents the direction of transcription. The T1SS and DVU1012 region of DvH (DVU1012 to DVU1020) was compared to the *lap* operon (PP0168 to PP0164) of *P. putida* (43). Genes of the same color are presumed orthologs. For select genes, the amino acid length for the encoded protein is displayed below the encoding *D. vulgaris* Hildenborough gene.

Such large microbial surface proteins have been grouped into seven categories based on sequence alignment and predicted motifs (24). Of these, only the LapA, AidA, and Bap families contain calcium-binding domains necessary for T1SS export. The LapA family is the only one to contain the von Willebrand factor A motif found in the protein encoded by DVU1012 and the glycine-rich domains found in both DVU1012 and DVU1545 proteins. The two *D. vulgaris* Hildenborough proteins also appear to be missing the conserved VCBS (from *Vibrio*, *Colwellia*, *Bradyrhizobium*, and *Shewanella*) repeat in the AidA family and cadherin motifs found in both the AidA and Bap families. Thus, based on the current categorization (24), *D. vulgaris* Hildenborough biofilm proteins appear to most closely resemble the large adhesion protein (Lap) system. The T1SS export system and surface proteins for *D. vulgaris* Hildenborough biofilm as well as their gene arrangement are similar (Fig. 6) to the Lap system, best studied in environmental pseudomonads *Pseudomonas putida* and *P. fluorescens* (25). DVU1017 is currently annotated as *rtxB* (repeat-in-toxin) based on similarity to the hemolysin export system of pathogens (26). This present study has established DVU1017 as essential for biofilm in *D. vulgaris* Hildenborough. Thus, it would be more congruous to rename this system as associated with biofilm formation. We propose that DVU1017 be renamed *lapB* in accordance with the naming system developed in the pseudomonads.

Furthermore, DVU1019 (Fig. 6) is annotated as encoding a transglutaminase-like cysteine protease and, based on synteny with the pseudomonad system, may serve a similar function as the cysteine protease LapG. In response to c-di-GMP, LapG is responsible in the pseudomonads for release of the adhesin LapA from the cell surface resulting in biofilm detachment (27–29). In the environmental pseudomonads, a GGDEF/EAL family protein, LapD, binds c-di-GMP, thereby modulating attachment to the substratum (29). Interestingly, DVU1020 shows synteny with *lapD* but encodes a PAS-fold-containing, HD domain sensory box protein, suggesting that *D. vulgaris* Hildenborough may have a different signaling mechanism regulating biofilm formation. The roles in *D. vulgaris* Hildenborough biofilm formation of the proteins encoded in the DVU1017 genome region warrant further inquiry.

In the Lap system of the environmental pseudomonads, the adhesins LapA and LapF perform noticeably different functions. LapA is required for stable attachment on abiotic surfaces, while LapF is not required for biofilm initiation but likely plays a role in cell-to-cell interactions during biofilm maturation (23, 30, 31). DVU1012 and DVU1545 mutants do not have the same phenotypes under the conditions tested. While LapA is required for biofilm formation in the environmental pseudomonads, neither DVU1012 nor DVU1545 is uniquely required for *D. vulgaris* Hildenborough biofilm under these reactor conditions (Fig. 4d). In the environmental pseudomonads, the genes encoding LapA and LapF are adjacent to their own T1SS transport machineries (31). DVU1545 does not appear to have its own export machinery as the only T1SS genes identified in the *D. vulgaris* Hildenborough genome are adjacent to DVU1012. If DVU1545 had a dedicated T1SS, the deletion of the ABC transporter DVU1017 would not be expected to inhibit export of the DVU1545 protein, but it does. DVU1012 was previously identified as an outer membrane protein in non-biofilm, batch cultures; however, DVU1545 was not (13). This suggests that under certain conditions, these two proteins may have distinctive roles that were not observed in the present study. Further characterization of DVU1012 and DVU1545 is under way.

Numerous studies have used DvH-MO-derived strains, and the results of those studies should be considered in the context of the known DvH-MO-specific mutations (see http://desulfovibriomaps.biochem.missouri.edu/mutants or contact the corresponding author for more information). Two published studies have used DvH-MO strains specifically for biofilm analyses (32, 33). The discovery of biofilm deficiency in DvH-MO described here may change the interpretation of the results in these past studies. In one, a DVU0636 transposon mutant, a c-di-GMP-modulating response regulator, was found to produce little to no biofilm (32). This could be due to the parental phenotype rather than the DVU0636 interruption. In the other, a DvH-MO *pil*A mutant was used as a non-biofilm-forming control strain in a *D. vulgaris* Hildenborough and methanogen coculture (33). This coculture was found to be unstable compared to the wild-type *D. vulgaris* Hildenborough and methanogen coculture because the cells completely washed out of the reactor in the effluent. In this study, DvH-MO would adequately represent a biofilm-deficient strain. However, the cause of the instability could be due to previously unidentified factors instead of, or in addition to, the lack of biofilm and role of pili in the interactions. These factors may include the lack of DVU1012 and DVU1545 on the *D. vulgaris* Hildenborough cell surface or possibly even an effect from another of the mutations in DvH-MO. Further investigation into whether these surface proteins play a role in *D. vulgaris* Hildenborough and methanogen interactions is needed.

The discovery of altered biofilm formation capacities in what were considered to be identical *D. vulgaris* Hildenborough strains has ultimately resulted in identification of genes required for biofilm formation. While this unintended laboratory-driven evolution was advantageous in elucidating these genes, it also serves as a cautionary tale. We do not know what the pressure was for losing the capacity to form biofilm by DvH-MO. Single-colony isolations for producing ~20 other mutants in the DvH-MT background have yet to result in a spontaneously biofilm-negative strain; thus, routine genetic manipulations do not appear to drive rapid biofilm loss. However, unrecognized laboratory selection, if not monitored, may lead to unexpected results and hinder reproducibility of experiments between laboratories. As next-generation sequencing becomes increasingly affordable, periodic resequencing of laboratory strains is no longer cost prohibitive. Conclusions can then be made within the context of all deviations in the strain, ultimately expanding the reproducibility and reliability of lab-to-lab comparisons.

## MATERIALS AND METHODS

### Strains, media, and growth conditions.

All strains used in this study are listed in Table S2 and are available upon request. DvH-MO and DvH-MT were both originally from the ATCC (29579; Manassas, VA), but have been maintained in separate laboratories at the University of Missouri and Montana State University. DvH-MO was received from the ATCC in 2003. DvH-MT was received at Montana State University from Terry C. Hazen in 2007 and was sent to the University of Missouri in 2013. Neither had been subjected to directed evolution, and stocks had been stored in 10 to 25% (vol/vol) glycerol solutions at −80°C. The intralaboratory lineage has not been determined, although it is likely that DvH-MO has gone through single-colony isolation.

*Escherichia coli* cultures were grown aerobically at 37°C in LC medium (per liter: 10 g tryptone, 5 g sodium chloride, 5 g yeast extract) or on LC agar plates (1.5% [wt/vol] agar) and recovered after transformation in modified SOC medium (0.5% [wt/vol] yeast extract, 0.9% [wt/vol] tryptone, 10 mM sodium chloride, 2.5 mM potassium chloride, 20 mM glucose, 10 mM magnesium chloride, 10 mM magnesium sulfate). Where indicated, kanamycin or spectinomycin was added to LC medium to a final concentration of 50 or 100 μg/ml, respectively (Gold Biotechnology, Inc., St. Louis, MO).

Unless otherwise specified, *D. vulgaris* Hildenborough was cultured in an anaerobic growth chamber (approximately 95% N_2_ and 5% H_2_; Coy Laboratory Products, Inc., Grass Lake, MI) in MO medium (34) supplemented with 1% (wt/vol) yeast extract, 60 mM sodium lactate, 30 mM sodium sulfate, 1.2 mM sodium thioglycolate as reductant, and 0.64 μM resazurin as a redox indicator (MOYLS4) (35). The pH was adjusted to 7.2 with hydrochloric acid. For growth on solidified media, *D. vulgaris* Hildenborough cultures were embedded in MOYLS4 solidified with 1.5% (wt/vol) agar by inoculating empty petri dishes, pouring molten agar over the inoculum, and swirling. When solidified, plates were inverted and incubated in AnaeroPack System rectangular jars containing sachets that absorb oxygen (Mitsubishi Gas, Chemical Co., Inc., Tokyo, Japan). DvH-MO and DvH-MT were cultured at 34 and 30°C, respectively, corresponding to the temperatures routinely used in each laboratory. Where indicated, 5-fluorouracil (5-FU; 40 μg/ml [Acros Organics, Geel, Belgium]), G418 (400 μg/ml; Gold Biotechnology, Inc.), or spectinomycin (100 μg/ml) was added to the medium. *D. vulgaris* Hildenborough cultures were routinely inoculated onto aerobic LC plates containing 40 mM glucose to ensure there was no aerobic contamination.

LS4D medium (pH 7.2) (modified from Borglin et al. [36] as described by De León et al. [37]) containing 60 mM sodium lactate and 50 mM sodium sulfate (in initial biofilm reactors) or 20 mM sodium lactate, 16.6 mM sodium sulfate, and 106 mM sodium chloride (in comparisons presented in Fig. 4) was used in the biofilm reactors and *D. vulgaris* Hildenborough cultures prepared for reactor inoculation (designated 60:50 LS4D and 20:16.6 LS4D, respectively). The purpose of the sodium chloride addition in the latter medium was to recuperate the sodium lost when sodium lactate and sodium sulfate were decreased. All LS4D cultures were incubated at 30°C.

### Biofilm reactors.

*D. vulgaris* Hildenborough biofilms were grown in anaerobic CDC biofilm reactors (BioSurface Technologies Corp., Bozeman, MT) containing seven Swiss glass microscope slides (Thermo Fisher Scientific, Waltham, MA) halved to be 7.6 cm long by 1.25 cm wide and cleaned with ethanol. These slides were attached to polypropylene coupon holders suspended from the lid that allow for temporal sampling of the biofilm. One sampling port contained a stopper and a plugged, 15.25-cm, 18-gauge septum penetration needle to allow for sampling of planktonic cells (Cadence, Inc., Cranston, RI). The reactors were maintained at 30°C and stirred at 80 rpm. A continuous flow of filtered high-purity 4.8-grade nitrogen gas was passed through the headspace of the reactor vessels at approximately 100 ml/min. Early DvH-MO and DvH-MT comparisons and reactor experiments for genomic sequencing (below) were performed in 60:50 LS4D medium. These conditions resulted in biofilms for which the maximum thickness was dictated by the limit of diffusion (33) and the optical density at 600 nm (OD_600_ [blanked on uninoculated LS4D here and throughout]) of the planktonic fraction was at or above 1.0. Subsequent analyses were performed in 20:16.6 LS4D medium. These reactors maintained a planktonic OD_600_ of 0.4 to 0.6 and were more stable, and the biofilms were limited by the reactor carrying capacity.

Approximately 30 h prior to reactor inoculation, 1-ml freezer stocks (10% [vol/vol] glycerol) of *D. vulgaris* Hildenborough were inoculated into 10 ml of LS4D with lactate-sulfate corresponding to reactor conditions. Reactors were inoculated with 10 ml of mid- to late-logarithmic cells at an OD_600_ of 0.8. After 24 h, when the planktonic OD_600_ was between 0.4 and 0.6, fresh anaerobic medium was pumped into the reactor at 0.70 ml/min for a dilution rate (*D*) of 0.112 h^−1^ (0.95 ml/min and *D* = 0.152 h^−1^ for the 60:50 LS4D runs; MasterFlex L/S Digital Drive with a standard pump head for L/S 13 tubing [Cole-Parmer, Vernon Hills, IL]).

Every 24 h, a biofilm coupon was removed from each reactor and replaced by a sterile stopper. The biofilm-coated slide was dipped into sterile 1× phosphate-buffered saline (PBS [pH 7.3]) (38) three times to remove planktonic and loosely associated cells. The biofilm was scraped into a 1.5-ml microcentrifuge tube with a sterile razor blade, and the slide and razor blade were rinsed with sterile PBS (1.5 ml total). These samples were centrifuged for 2 min at 16,000 × *g*, the supernatant was removed, and the pellets were frozen at −20°C until processed. Biofilm samples were resuspended in 1 ml sterile water, and biomass was measured as protein by Bradford assay with bovine serum albumin (BSA [Thermo Fisher]) as a standard (39).

In addition, every 24 h a planktonic sample was collected from each reactor. With a sterile syringe, 4.5 ml of planktonic culture was removed through the 15.25-cm needle, of which 3 ml was placed in a Balch tube for OD_600_ measurement and 1.5 ml was collected in a microcentrifuge tube for protein and DNA analyses. The samples in these tubes were pelleted for 2 min at 16,000 × *g*. The supernatant was removed and applied to pH paper to ensure the pH was not changing in the reactors. The pellets were frozen at −20 °C. At either 72 or 96 h and at the end of the reactor run, an additional 0.5 ml of planktonic cells was removed from each reactor and spread onto LC-glucose plates at 30°C to test for aerobic contamination of the reactors.

Three reactors were operated simultaneously from the same medium reservoir. For all reactor experiments with mutant strains, the DvH-MT parental strain, JWT700, was grown concurrently as a control and to track consistency across reactor experiments (Fig. 4; Fig. S1). Most mutants were grown in two separate reactor runs, varying which of the three reactors contained the mutant to account for any differences in the individual reactor systems. The genotype of each strain was reconfirmed by PCR at the end of each reactor run with gene-specific primers and with primers specific to the JW710 (DvH-MO) and JWT700 (DvH-MT) barcodes (see Table S3 in the supplemental material). Furthermore, the genotype of cells in reactor runs of the DVU1017 complement construct or site-directed mutants was confirmed by sequencing the DVU1017 region. All confirmations were performed on the biofilm for biofilm-producing strains and on the planktonic fraction for biofilm-negative strains.

10.1128/mBio.01696-17.4 TABLE S3  Primers used in this study. Download TABLE S3, XLS file, 0.1 MB. Copyright © 2017 De León et al. 2017 De León et al. This content is distributed under the terms of the Creative Commons Attribution 4.0 International license.

### Batch biofilms.

Duplicate anaerobic Balch tubes containing halved glass microscope slides submerged in 5 ml 60:50 LS4D were inoculated with 0.1 ml of a mid-logarithmic culture and incubated at 30°C for 48 h. Simultaneously, Balch tubes containing 60:50 LS4D but lacking a glass slide were inoculated, and OD_600_ was measured for 48 h to test whether growth rates were similar between strains. After 48 h, batch biofilm tubes were destructively sampled, the slides were rinsed and scraped, and protein was quantified as described for biofilm reactor slides (described above).

### Genome sequencing and analysis.

Genomic DNA (gDNA) was extracted with the Wizard Genomic DNA purification kit (Promega Corp., Madison, WI) per the manufacturer’s guidelines for Gram-negative bacteria. The extracted DNA was resuspended in 50 μl of sterile 10 mM Tris-HCl (pH 8.5) for genomic sequencing and 50 μl of sterile water for other downstream analyses (e.g., Southern blots and PCR). For genome resequencing, gDNA was extracted from 3 ml of DvH-MO or DvH-MT cultures grown to an OD_600_ of 0.9 in Balch tubes containing 10 ml 60:50 LS4D at 30°C. In addition, DvH-MO from the same set of freezer stocks as the batch culture inoculum was grown in the biofilm reactors. In this case, no biofilm sample was collected until 96 h. Instead, at 168 h of continuous flow, two biofilm samples of DvH-MO were collected (one for gDNA extraction and one for protein) and the reactor continued for 192 h to confirm that the biofilm was at steady state when the sample was collected for sequencing. Genomic DNA was quantified in a Qubit fluorometer with the double-stranded DNA (dsDNA) BR assay kit (Thermo Fisher Scientific). Samples were prepared, barcoded, and sequenced on an Illumina MiSeq (2 × 150 bases; Illumina, Inc., San Diego, CA) at the University of Missouri DNA Core (http://dnacore.missouri.edu/).

Raw Illumina sequences were mapped to the published *D. vulgaris* Hildenborough genome (15) with Bowtie 2 (40) within Geneious (v.8.1.8; Biomatters, Ltd., Auckland, New Zealand). True variations in nucleotide sequence were defined as deviations from the published sequence where at least 10% of the reads covering the region contained the deviation and, if the frequency was less than 80%, the strand bias was limited to less than 70%. These variants could include SNPs, insertions, or deletions.

### Plasmid and strain construction.

All of the plasmids and strains used in this study are listed in Table S2 and are available upon request. Primers utilized in this study are listed in Table S3. All plasmids were constructed via sequence and ligation-independent cloning (SLIC) (41). Briefly, genomic, plasmid backbone, and antibiotic resistance cassette fragments were PCR amplified with Herculase II fusion DNA polymerase (Agilent Technologies, Santa Clara, CA) with primers from Integrated DNA Technologies, Inc. (Coralville, IA). Residual primers and enzyme were removed from all amplicons with the Wizard SV gel and PCR cleanup system (Promega Corp.). Where necessary, PCR products were gel extracted with this Wizard system to remove secondary amplification products. PCR products from plasmid templates were treated with DpnI for 1 h per the manufacturers’ guidelines to degrade methylated template DNA (New England Biolabs [NEB], Ipswich, MA). All PCR fragments were combined and treated with T4 DNA polymerase (NEB) as instructed by Li and Elledge (41) for the SLIC reaction, and 5 μl of these treated fragments was transformed into 50 μl α-select, silver-efficiency *E. coli* (Bioline, London, United Kingdom) per the manufacturers’ guidelines, except that recovery was in 195 μl of SOC medium. Recovered cells were plated on solidified LC medium containing kanamycin or spectinomycin as appropriate. Colonies were screened for the correct insert size with *Taq* DNA polymerase (NEB), and those with the correct size were inoculated into 5 ml LC containing the appropriate antibiotic. Plasmid DNA was prepared from 1.5 ml *E. coli* transformants with the GeneJET plasmid miniprep kit, quantified by Qubit (Thermo Fisher Scientific), and verified by sequencing. All plasmids generated in this study were unable to replicate in *D. vulgaris* Hildenborough.

The use of a *D. vulgaris* Hildenborough parental strain lacking the uracil phosphoribosyltransferase gene (*upp* [pyrimidine salvage pathway]) allowed for counterselection with the toxic pyrimidine 5-fluorouracil (5-FU). In DvH-MO, this strain was previously constructed by replacing *upp* with a unique 40-bp barcode (5′ ACG TGC CTA AAG TGT ACT ACT GAC ACC TCC GCG ATG AGT A 3′) for strain identification (1). To generate a parental Δ*upp* strain in DvH-MT, a plasmid that contained a unique 39-bp barcode (5′ TAC GTT ACC GTT ACT CAG TCA GTA CCA CAA CTC AGA CGT 3′) flanked by *D. vulgaris* Hildenborough sequences upstream and downstream of the gene *upp* (DVU1025) was electroporated into DvH-MT wild-type cells and selected for 5-FU resistance, as described previously (1). After phenotypic confirmation for 5-FU resistance and spectinomycin and G418 sensitivity, as well as genome structure by Southern blot, one isolate was designated JWT700.

To create site-specific mutations or in-frame deletions, the gene of interest (DVU1012, DVU1017, or DVU1545) in the Δ*upp* parental strain was first replaced with the antibiotic resistance cassette encoding the aminoglycoside-3′-phosphotransferase (*npt* conferring Km^r^) driven by the native promoter (P_*npt*_) followed by the *upp* gene (P*_npt_-npt-upp*) as previously described (42). These marker-exchange strains (G418^r^ 5-FU^s^) were the parental strains for subsequent removal of the marker resulting in in-frame deletion strains. Furthermore, the marker-exchange strain of the DVU1017 ATP-binding domain region (deletion of 1,543 to 2,331 bp and G1542T) was the parental strain for complementation by replacing the cassette with the wild-type gene segment and for creation of site-specific mutations by introduction of the altered gene (e.g., mutations resulting in A635P and A635L in the DVU1017 protein). These constructs were accomplished by selection for 5-FU resistance as previously described (35). After antibiotic phenotypic confirmation (as described for JWT700 in the previous paragraph), in-frame deletion strains were confirmed by Southern blot. Complement and mutated gene strains were confirmed by sequencing.

To differentiate complement and SNP strains from “wild-type” strains of DVU1017, the stop codon of these constructed strains was changed from TAG to TGA (41.8% and 45.3% codon usage on chromosome, respectively), eliminating an FspBI cut site. PCR amplification with DVU1017-1800F and DVU1017-dnR followed by FspBI (Thermo Fisher Scientific) digestion per the manufacturer’s guidelines was performed routinely to ensure no detectable mixing of strains.

Attempts to generate the complement or SNP plasmids containing the entire DVU1017 gene in *E. coli* proved problematic. Colonies were only formed when recovery and growth were carried out at 30°C. PCR screening and sequencing consistently identified mutations in either the peptidase or ATP-binding domains within the gene and included amino acid changes, frameshifts, and a transposase insertion. To circumvent this apparent negative selection in *E. coli*, a marker-exchange mutant was created that replaced only the ATP-binding domain region of DVU1017 (see above and Table S2). This allowed for complement and SNP plasmid construction without the necessity of a complete copy of the gene within *E. coli*. To prevent possible polar effects of the partial DVU1017 gene copy within *D. vulgaris* Hildenborough, a stop codon was introduced at the point of deletion for the marker-exchange and markerless deletion strains. This stop codon was repaired during complementation and SNP introduction.

### Preparation of antibodies.

Sequence analysis, model building, and cell surface proteolysis experiments were used to identify regions of *D. vulgaris* Hildenborough DVU1012 and DVU1545 proteins useful for the preparation of antibodies targeting surface-accessible sites. Candidate amino acid sequences were assessed for solubility, antigenicity, and surface probability, among other characteristics, with proprietary software (OptimumAntigen; GenScript, Piscataway, NJ) and codon optimized. Peptides for the target regions (LGTLDPLVFERGTEC for DVU1012, GLESPTSAQESDGAC for DVU1545) were synthesized and used to obtain polyclonal antibodies (rabbit), which in turn were affinity purified against these peptides (GenScript).

### Immunogold labeling of cells and electron microscopy.

As an initial wash step, *D. vulgaris* Hildenborough cell pellet aliquots (0.1 g) of the DvH-MT parental strain (JWT700), the DVU1017 deletion mutant (JWT704), and the DVU1012 and DVU1545 double-deletion mutant (JWT709) were suspended in 1 ml PBS and centrifuged (2,000 × *g* for 5 min). To improve contrast in images of cell surface-bound antibodies, washed cell pellets were resuspended in 1 ml of 2 mM MgCl_2_, lysed via freeze-thaw, and treated with DNase I (5 μl of 1-mg/ml stock per ml sample; bovine pancreas [Sigma-Aldrich, St. Louis, MO]) for 15 min at room temperature. Treated cell suspensions were centrifuged (2,000 × *g* for 5 min), and cell pellets were resuspended in distilled water (dH_2_O). Samples from these suspensions were diluted 1:10 in dH_2_O prior to electron microscopy processing. For immunolabeling, all steps were conducted at room temperature. To begin, 4 μl of diluted cells was placed on glow-discharged electron microscope grids (Formvar/carbon film, 200 mesh copper; Electron Microscopy Sciences, Hatfield, PA) and left for 10 min prior to blotting the remaining liquid to about 1 μl. These grids were placed sample side down on 70-μl drops of blocking solution (20 mM Tris-HCl [pH 7.5], 150 mM NaCl, and either 0.1% bovine serum albumin [BSA] or 0.5% nonfat powdered milk) situated on a sheet of paraffin film (Parafilm M). After 30 min, grids were transferred (sample side down) to neighboring 70-μl drops containing the primary antibody diluted 1:100 in blocking solution. Following a 60-min incubation period, grids were moved to fresh 70-μl drops of blocking solution for 15 min prior to being transferred to 70-μl drops of secondary antibody (anti-rabbit IgG coupled to 10-nm-diameter colloidal gold; Sigma-Aldrich) diluted 1:50 in blocking solution. After 30 min, grids were transferred to 70-μl blocking solution drops twice, for 5 min each time, followed by sequential transfer to three dH_2_O drops for 5 min each. Following the last rinse, grids were turned sample side up and blotted to leave about 1 μl of liquid. To the remaining liquid, 2 μl of 2% (wt/vol) uranyl acetate was added, which after 1 min was blotted to dryness. Negatively stained grids were examined in an FEI CM200 electron microscope operating at 200 kV and equipped with a Gatan digital camera. Micrographs were recorded over a magnification range of 10,000× to 30,000×.

### Accession number(s).

The *D. vulgaris* Hildenborough genome resequencing data have been deposited in the NCBI Sequence Read Archive under accession no. SRR5780888 to SRR5780890.

## References

[1] Keller KL, Bender KS, Wall JD 2009 Development of a markerless genetic exchange system for *Desulfovibrio vulgaris* Hildenborough and its use in generating a strain with increased transformation efficiency. Appl Environ Microbiol 75:7682–7691. doi:10.1128/AEM.01839-09. 19837844 PMC2794091

[2] Hulshoff Pol LW, Lens PNL, Stams AJM, Lettinga G 1998 Anaerobic treatment of sulphate-rich wastewaters. Biodegradation 9:213–224. doi:10.1023/A:1008307929134. 10022065

[3] Chardin B, Dolla A, Chaspoul F, Fardeau ML, Gallice P, Bruschi M 2002 Bioremediation of chromate: thermodynamic analysis of the effects of Cr(VI) on sulfate-reducing bacteria. Appl Microbiol Biotechnol 60:352–360. doi:10.1007/s00253-002-1091-8. 12436319

[4] Chakraborty R, Wu CH, Hazen TC 2012 Systems biology approach to bioremediation. Curr Opin Biotechnol 23:483–490. doi:10.1016/j.copbio.2012.01.015. 22342400

[5] Cooney MJ, Roschi E, Marison IW, Comninellis C, von Stockar U 1996 Physiologic studies with the sulfate-reducing bacterium *Desulfovibrio desulfuricans*: evaluation for use in a biofuel cell. Enzyme Microb Technol 18:358–365. doi:10.1016/0141-0229(95)00132-8. 8882004

[6] Javaherdashti R 2011 Impact of sulphate-reducing bacteria on the performance of engineering materials. Appl Microbiol Biotechnol 91:1507–1517. doi:10.1007/s00253-011-3455-4. 21786108

[7] Zhang P, Xu D, Li Y, Yang K, Gu T 2015 Electron mediators accelerate the microbiologically influenced corrosion of 304 stainless steel by the *Desulfovibrio vulgaris* biofilm. Bioelectrochemistry 101:14–21. doi:10.1016/j.bioelechem.2014.06.010. 25023048

[8] Qi Z, Chen L, Zhang W 2016 Comparison of transcriptional heterogeneity of eight genes between batch *Desulfovibrio vulgaris* biofilm and planktonic culture at a single-cell level. Front Microbiol 7:597. doi:10.3389/fmicb.2016.00597. 27199927 PMC4847118

[9] Zhang W, Culley DE, Nie L, Scholten JCM 2007 Comparative transcriptome analysis of *Desulfovibrio vulgaris* grown in planktonic culture and mature biofilm on a steel surface. Appl Microbiol Biotechnol 76:447–457. doi:10.1007/s00253-007-1014-9. 17571259

[10] Clark ME, Edelmann RE, Duley ML, Wall JD, Fields MW 2007 Biofilm formation in *Desulfovibrio vulgaris* Hildenborough is dependent upon protein filaments. Environ Microbiol 9:2844–2854. doi:10.1111/j.1462-2920.2007.01398.x. 17922767

[11] Clark ME, He Z, Redding AM, Joachimiak MP, Keasling JD, Zhou JZ, Arkin AP, Mukhopadhyay A, Fields MW 2012 Transcriptomic and proteomic analyses of *Desulfovibrio vulgaris* biofilms: carbon and energy flow contribute to the distinct biofilm growth state. BMC Genomics 13:138. doi:10.1186/1471-2164-13-138. 22507456 PMC3431258

[12] Whittaker CA, Hynes RO 2002 Distribution and evolution of von Willebrand/integrin A domains: widely dispersed domains with roles in cell adhesion and elsewhere. Mol Biol Cell 13:3369–3387. doi:10.1091/mbc.E02-05-0259. 12388743 PMC129952

[13] Walian PJ, Allen S, Shatsky M, Zeng L, Szakal ED, Liu H, Hall SC, Fisher SJ, Lam BR, Singer ME, Geller JT, Brenner SE, Chandonia JM, Hazen TC, Witkowska HE, Biggin MD, Jap BK 2012 High-throughput isolation and characterization of untagged membrane protein complexes: outer membrane complexes of *Desulfovibrio vulgaris*. J Proteome Res 11:5720–5735. doi:10.1021/pr300548d. 23098413 PMC3516867

[14] Han BG, Dong M, Liu H, Camp L, Geller J, Singer M, Hazen TC, Choi M, Witkowska HE, Ball DA, Typke D, Downing KH, Shatsky M, Brenner SE, Chandonia JM, Biggin MD, Glaeser RM 2009 Survey of large protein complexes in *D. vulgaris* reveals great structural diversity. Proc Natl Acad Sci U S A 106:16580–16585. doi:10.1073/pnas.0813068106. 19805340 PMC2742403

[15] Heidelberg JF, Seshadri R, Haveman SA, Hemme CL, Paulsen IT, Kolonay JF, Eisen JA, Ward N, Methe B, Brinkac LM, Daugherty SC, Deboy RT, Dodson RJ, Durkin AS, Madupu R, Nelson WC, Sullivan SA, Fouts D, Haft DH, Selengut J, Peterson JD, Davidsen TM, Zafar N, Zhou L, Radune D, Dimitrov G, Hance M, Tran K, Khouri H, Gill J, Utterback TR, Feldblyum TV, Wall JD, Voordouw G, Fraser CM 2004 The genome sequence of the anaerobic, sulfate-reducing bacterium *Desulfovibrio vulgaris* Hildenborough. Nat Biotechnol 22:554–559. doi:10.1038/nbt959. 15077118

[16] Delepelaire P 2004 Type I secretion in Gram-negative bacteria. Biochim Biophys Acta 1694:149–161. doi:10.1016/j.bbamcr.2004.05.001. 15546664

[17] Buchan DWA, Minneci F, Nugent TCO, Bryson K, Jones DT 2013 Scalable web services for the PSIPRED protein analysis workbench. Nucleic Acids Res 41:W349–W357. doi:10.1093/nar/gkt381. 23748958 PMC3692098

[18] Cordes FS, Bright JN, Sansom MSP 2002 Proline-induced distortions of transmembrane helices. J Mol Biol 323:951–960. doi:10.1016/S0022-2836(02)01006-9. 12417206

[19] Cypionka H 1989 Characterization of sulfate transport in *Desulfovibrio desulfuricans*. Arch Microbiol 152:237–243. doi:10.1007/BF00409657. 2476099

[20] Drake JW 1991 A constant rate of spontaneous mutation in DNA-based microbes. Proc Natl Acad Sci U S A 88:7160–7164. doi:10.1073/pnas.88.16.7160. 1831267 PMC52253

[21] Herring CD, Raghunathan A, Honisch C, Patel T, Applebee MK, Joyce AR, Albert TJ, Blattner FR, van den Boom D, Cantor CR, Palsson BØ 2006 Comparative genome sequencing of *Escherichia coli* allows observation of bacterial evolution on a laboratory timescale. Nat Genet 38:1406–1412. doi:10.1038/ng1906. 17086184

[22] Finkel SE, Kolter R 1999 Evolution of microbial diversity during prolonged starvation. Proc Natl Acad Sci U S A 96:4023–4027. doi:10.1073/pnas.96.7.4023. 10097156 PMC22413

[23] Hinsa SM, Espinosa-Urgel M, Ramos JL, O’Toole GA 2003 Transition from reversible to irreversible attachment during biofilm formation by *Pseudomonas fluorescens* WCS365 requires an ABC transporter and a large secreted protein. Mol Microbiol 49:905–918. doi:10.1046/j.1365-2958.2003.03615.x. 12890017

[24] Yousef F, Espinosa-Urgel M 2007 *In silico* analysis of large microbial surface proteins. Res Microbiol 158:545–550. doi:10.1016/j.resmic.2007.04.006. 17576051

[25] Berne C, Ducret A, Hardy GG, Brun YV 2015 Adhesins involved in attachment to abiotic surfaces by Gram-negative bacteria, p 163–199. *In* Ghannoum M, Parsek M, Whiteley M, Mukherjee PK (ed), Microbial biofilms, 2nd ed. ASM Press, Washington, DC. 10.1128/microbiolspec.MB-0018-2015 PMC4566860 26350310

[26] Dehal PS, Joachimiak MP, Price MN, Bates JT, Baumohl JK, Chivian D, Friedland GD, Huang KH, Keller K, Novichkov PS, Dubchak IL, Alm EJ, Arkin AP 2010 MicrobesOnline: an integrated portal for comparative and functional genomics. Nucleic Acids Res 38:D396–D400. doi:10.1093/nar/gkp919. 19906701 PMC2808868

[27] Newell PD, Monds RD, O’Toole GA 2009 LapD is a bis-(3′,5′)-cyclic dimeric GMP-binding protein that regulates surface attachment by *Pseudomonas fluorescens* Pf0-1. Proc Natl Acad Sci U S A 106:3461–3466. doi:10.1073/pnas.0808933106. 19218451 PMC2651287

[28] Gjermansen M, Nilsson M, Yang L, Tolker-Nielsen T 2010 Characterization of starvation-induced dispersion in *Pseudomonas putida* biofilms: genetic elements and molecular mechanisms. Mol Microbiol 75:815–826. doi:10.1111/j.1365-2958.2009.06793.x. 19602146

[29] Newell PD, Boyd CD, Sondermann H, O’Toole GA 2011 A c-di-GMP effector system controls cell adhesion by inside-out signaling and surface protein cleavage. PLoS Biol 9:e1000587. doi:10.1371/journal.pbio.1000587. 21304920 PMC3032545

[30] Espinosa-Urgel M, Salido A, Ramos JL 2000 Genetic analysis of functions involved in adhesion of *Pseudomonas putida* to seeds. J Bacteriol 182:2363–2369. doi:10.1128/JB.182.9.2363-2369.2000. 10762233 PMC111295

[31] Martínez-Gil M, Yousef-Coronado F, Espinosa-Urgel M 2010 LapF, the second largest *Pseudomonas putida* protein, contributes to plant root colonization and determines biofilm architecture. Mol Microbiol 77:549–561. doi:10.1111/j.1365-2958.2010.07249.x. 20545856

[32] Rajeev L, Luning EG, Altenburg S, Zane GM, Baidoo EEK, Catena M, Keasling JD, Wall JD, Fields MW, Mukhopadhyay A 2014 Identification of a cyclic-di-GMP-modulating response regulator that impacts biofilm formation in a model sulfate reducing bacterium. Front Microbiol 5:382. doi:10.3389/fmicb.2014.00382. 25120537 PMC4114195

[33] Brileya KA, Camilleri LB, Zane GM, Wall JD, Fields MW 2014 Biofilm growth mode promotes maximum carrying capacity and community stability during product inhibition syntrophy. Front Microbiol 5:693. doi:10.3389/fmicb.2014.00693. 25566209 PMC4266047

[34] Zane GM, Yen HC, Wall JD 2010 Effect of the deletion of *qmoABC* and the promoter-distal gene encoding a hypothetical protein on sulfate reduction in *Desulfovibrio vulgaris* Hildenborough. Appl Environ Microbiol 76:5500–5509. doi:10.1128/AEM.00691-10. 20581180 PMC2918943

[35] Christensen GA, Zane GM, Kazakov AE, Li X, Rodionov DA, Novichkov PS, Dubchak I, Arkin AP, Wall JD 2015 Rex (encoded by DVU_0916) in Desulfovibrio vulgaris Hildenborough is a repressor of sulfate adenylyl transferase and is regulated by NADH. J Bacteriol 197:29–39. doi:10.1128/JB.02083-14. 25313388 PMC4288696

[36] Borglin S, Joyner D, Jacobsen J, Mukhopadhyay A, Hazen TC 2009 Overcoming the anaerobic hurdle in phenotypic microarrays: generation and visualization of growth curve data for *Desulfovibrio vulgaris* Hildenborough. J Microbiol Methods 76:159–168. doi:10.1016/j.mimet.2008.10.003. 18996155

[37] Bowen De León KB, Young ML, Camilleri LB, Brown SD, Skerker JM, Deutschbauer AM, Arkin AP, Fields MW 2012 Draft genome sequence of *Pelosinus fermentans* JBW45, isolated during in situ stimulation for Cr(VI) reduction. J Bacteriol 194:5456–5457. doi:10.1128/JB.01224-12. 22965085 PMC3457247

[38] Ausubel FM, Brent R, Kingston RE, Moore DD, Seidman JG, Smith JA, Struhl K 1994 Current protocols in molecular biology, vol 2 John Wiley & Sons, New York, NY.

[39] Bradford MM 1976 A rapid and sensitive method for the quantitation of microgram quantities of protein utilizing the principle of protein-dye binding. Anal Biochem 72:248–254. doi:10.1016/0003-2697(76)90527-3. 942051

[40] Langmead B, Salzberg SL 2012 Fast gapped-read alignment with Bowtie 2. Nat Methods 9:357–359. doi:10.1038/nmeth.1923. 22388286 PMC3322381

[41] Li MZ, Elledge SJ 2007 Harnessing homologous recombination *in vitro* to generate recombinant DNA via SLIC. Nat Methods 4:251–256. doi:10.1038/nmeth1010. 17293868

[42] Parks JM, Johs A, Podar M, Bridou R, Hurt RA Jr, Smith SD, Tomanicek SJ, Qian Y, Brown SD, Brandt CC, Palumbo AV, Smith JC, Wall JD, Elias DA, Liang L 2013 The genetic basis for bacterial mercury methylation. Science 339:1332–1335. doi:10.1126/science.1230667. 23393089

[43] Nelson KE, Weinel C, Paulsen IT, Dodson RJ, Hilbert H, Martins dos Santos VAP, Fouts DE, Gill SR, Pop M, Holmes M, Brinkac L, Beanan M, DeBoy RT, Daugherty S, Kolonay J, Madupu R, Nelson W, White O, Peterson J, Khouri H, Hance I, Lee PC, Holtzapple E, Scanlan D, Tran K, Moazzez A, Utterback T, Rizzo M, Lee K, Kosack D, Moestl D, Wedler H, Lauber J, Stjepandic D, Hoheisel J, Straetz M, Heim S, Kiewitz C, Eisen JA, Timmis KN, Düsterhöft A, Tümmler B, Fraser CM 2002 Complete genome sequence and comparative analysis of the metabolically versatile *Pseudomonas putida* KT2440. Environ Microbiol 4:799–808. doi:10.1046/j.1462-2920.2002.00366.x. 12534463

