# Supplementary material for: Unintended Laboratory-Driven Evolution Reveals Genetic Requirements for Biofilm Formation by Desulfovibrio vulgaris Hildenborough
Source: mBio. 2017 Oct 17;8(5):e01696-17. doi: 10.1128/mBio.01696-17 (PMC5646257; doi:10.1128/mBio.01696-17)
Supplement: TABLE S1 [file mbo005173543st1.doc]

**Table S1.** Deviations from published sequence in both DvH-MO and DvH-MT are possible sequencing errors in the original sequencing

| **Location^a^ (P-on plasmid)** | **Locus Tag** | **Nucleotide Change** | **Amino Acid Change** |
| --- | --- | --- | --- |
| 42,868 |  | +G |  |
| 211,390 |  | +A |  |
| 882,512 |  | (C)6 → (C)5 |  |
| 1,073,326 |  | C → T | E → K |
| 1,144,621 |  | +C |  |
| 1,191,167 | DVU1087 | (C)3 → (C)2 |  |
| 1,313,342 |  | +C |  |
| 1,363,430 | DVU1272 | C → A | R → I |
| 1,773,256 | DVU1698 | G → C | Q → E |
| 1,773,345 | DVU1698 | (A)3 → (A)2 | Frameshift |
| 1,897,097 |  | +T |  |
| 1,913,197 | DVU1842 | T → G | M → L |
| 2,083,306 |  | (C)3 → (C)4 |  |
| 2,381,876 | DVU2287 | T → G | U → G |
| 2,669,556 | DVU2557 | A → C | V → G |
| 2,982,789 |  | +CC |  |
| 3,056,213 |  | +G |  |
| 3,064,064 |  | CG → GC |  |
| 3,066,927 |  | +ACG |  |
| 3,140,577 | DVU3022 | -CA |  |
| 3,140,595 | DVU3022 | +GA |  |
| 3,142,198 | DVU3023 | A → G | D → G |
| 3,276,861 | DVU3129 | (C)4 → (C)5 |  |
| 3,443,816 | DVU3268-DVU3274 | +7622 bp Tandem Duplication | Tandem Duplication |
| 3,455,728 | DVU3280 | +T |  |
| 3,457,156 |  | +CGC |  |
| P_30,837 | DVUA0025 | +A |  |
| P_49,089 |  | G → C |  |
| P_99,978 | DVUA0073 | -CGTAGTGGAACGGCTTGACGC | GVKPFHYA → A |

^a^Reference sequence NCBI Accession No. NC_002937.3 and NC_005863.1
