# Supplementary figures and images for: Unintended Laboratory-Driven Evolution Reveals Genetic Requirements for Biofilm Formation by Desulfovibrio vulgaris Hildenborough
Source: mBio. 2017 Oct 17;8(5):e01696-17. doi: 10.1128/mBio.01696-17 (PMC5646257; doi:10.1128/mBio.01696-17)

**a**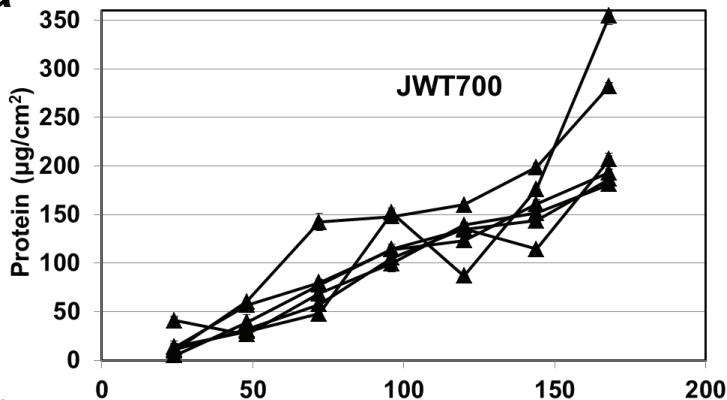**b**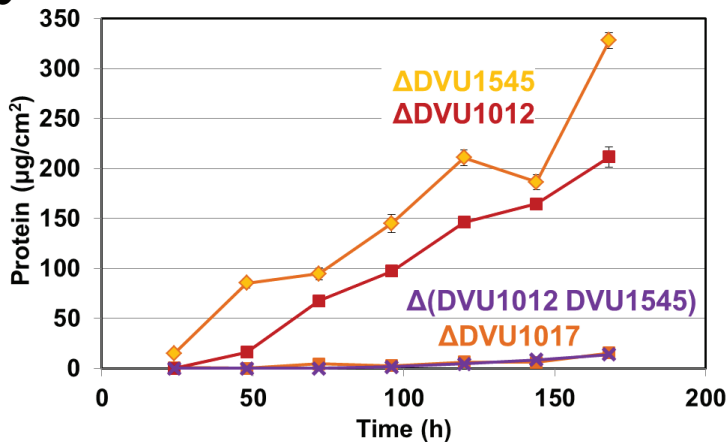

Supplement: FIG S1 [file mbo005173543sf1.pdf]
